# Manufacturing and Recycling Impact on Environmental Life Cycle Assessment of Innovative Wind Power Plant Part 2/2

**DOI:** 10.3390/ma14010204

**Published:** 2021-01-04

**Authors:** Patrycja Bałdowska-Witos, Krzysztof Doerffer, Michał Pysz, Piotr Doerffer, Andrzej Tomporowski, Marek Opielak

**Affiliations:** 1 Department of Technical Systems Engineering, Faculty of Mechanical Engineering, University of Science and Technology in Bydgoszcz, 85-796 Bydgoszcz, Poland; patrycja.baldowska-witos@utp.edu.pl (P.B.-W.); a.tomporowski@utp.edu.pl (A.T.); 2 Department of Manufacturing and Production Engineering, Faculty of Mechanical Engineering, Gdansk University of Technology, 80-233 Gdańsk, Poland; 3 Department of Energy and Industrial Apparatus, Faculty of Mechanical Engineering, Gdansk University of Technology, 80-233 Gdańsk, Poland; michal.pysz@pg.edu.pl; 4 Centre of Flow and Combustion, The Szewalski Institute of Fluid-Flow Machinery Polish Academy of Sciences, 80-231 Gdańsk, Poland; doerffer@imp.gda.pl; 5 Department of Transport, Combustion Engines and Ecology, Faculty of Mechanical Engineering, Lublin University of Technology, 20-618 Lublin, Poland; m.opielak@pollub.pl

**Keywords:** Eco-indicator 99, LCA, wind energy, recycling, innovative wind farm

## Abstract

The process of conversion of wind kinetic energy into electricity in innovative wind power plant emits practically no harmful substances into the environment. However, the production stage of its components requires a lot of energy and materials. The biggest problem during production planning process of an innovative wind power plant is selection of materials and technologies and, consequently, the waste generated at this stage. Therefore, the aim of this publication was to conduct an environmental analysis of the life cycle of elements of a wind turbine by means of life cycle assessment (LCA) method. The object of the research was a wind power plant divided into five sets of components (tower, turbine structure, rotors, generators, and instrumentation), made mainly of steel and small amounts of polymer materials. Eco-indicator 99 was used as an analytical procedure. The impact of the subjects of analysis on human health, ecosystem quality and resources was assessed. Among the analyzed components, the highest level of negative impact on the environment was characterized by the life cycle of the wind turbine tower. The application of recycling processes is reducing the negative impact on the environment in the perspective of the entire life cycle of all studied elements of the wind power plant construction.

## 1. Introduction

Human productive activity consists of using the natural resources of the surrounding nature. Simultaneously the same activity is a source of emission of pollutants, which are generated during the process of transforming natural resources. Buildings, machines, useful materials, energy sources are just a few examples.

Environmental factors such as dust and noise have a noticeable impact on people’s quality of life but they are not necessarily direct and significant threats to human health. The damage to the environment in all its aspects [1,2,3] is increasingly perceptible in the intensive exploitation of natural resources and the release of pollutants from industrial production of energy generating products or services.

Conventional energy uses huge amounts of fossil fuels (e.g., coal, oil, and gas). The largest share in the emission of environmental pollutants has been the production sector. Many manufacturing processes in this sector use fossil fuels. The release of pollutants into the environment takes place mainly in the processes of obtaining conventional fuels and their combustion [4].

**Figure 1:**
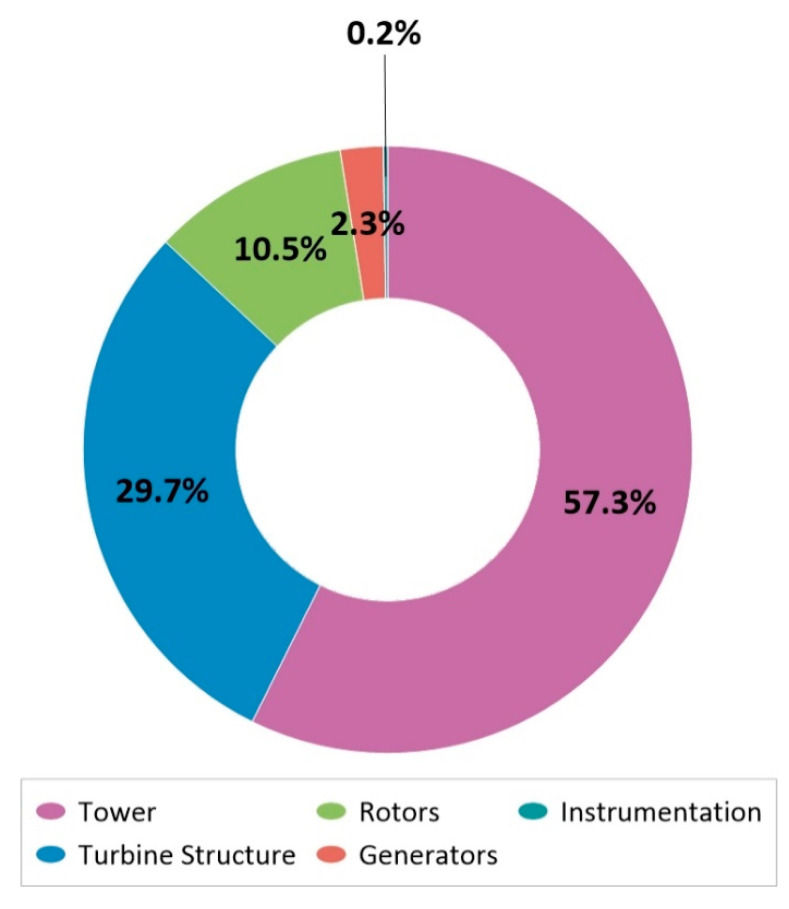
Mass participation of structure components of innovative wind farm.

One of the assumptions of sustainable development is to obtain clean energy. Wind energy is considered as such. However, despite its much lower carbon footprint, it cannot be regarded as completely clean because, just like traditional fossil fuel power plants, it emits CO2 and other pollutants and waste during construction, maintenance and disposal. The innovative 15 kW wind power plant is mainly made of steel. The weight distribution of an innovative wind power plant is shown in Figure 1 [5,6,7].

In the recent past, the awareness connected with environmental issues significantly increased. Many direct actions towards these issues were taken. Humans realized that the problem is much more complicated and an holistic approach is needed. The effort to minimize environmental impacts is especially connected with economic decisions that are often not fully justified economically. The whole area of economic activity requires consideration in the process of calculating the environmental impact of products and services. Companies, among others there are ones producing wind power plants, in these dynamically developing changes in the philosophy of economic and ecological activity and fulfilling social expectations in their life cycle (LCT), apart from construction and production aspects, are beginning to take environmental protection into account. The realization of this demand and achievement of the goal of maximizing environmental protection is made possible by a methodical tool, which is life cycle assessment (LCA) [8,9]. This methodology covers the whole life cycle of a product and enables its ecological evaluation. Thanks to the implementation of ISO standards, the LCA methodology is nowadays an assessment tool which ensures comparable analysis results while considering many important environmental problems. It provides an orderly approach when considering complex issues related to environmental impact assessment, taking into account the entire life cycle of research subjects. LCA is widely recognized as an effective tool for assessing the environmental impact of objects [1,10].

It is possible for each product and service to have a multifaceted impact on the environment. At different time intervals in different stages of its life cycle, with fluctuating intensity, they may also have varying ranges of impact [8,9,10,11]. Thus, such impact can be observed in one or more life stages from the supply of raw materials via production and distribution to operation along with disposal. Ecological analyses are carried out in order to answer the question of what is the potential damage caused to the environment as a result of the object’s life cycle. If the analysis does not fully answer such a question, it shall at least point out the potential environmental risks that can be expected as a result of the life cycle of the object. To achieve this, it is necessary to link the processes taking place at each stage of the life cycle with the environmental damage they cause. In particular in environmental protection areas, which include human and animal health, environmental quality, protection of agricultural land and forests, natural resources, or anthropogenic products [8,12,13].

Recent studies related to the manufacture, operation and management of wind farms drew special attention to the aspects of environmental protection. Life cycle assessment studies of wind farms [14,15] focused mainly on the comparative assessment of selected research objects and their impact on the condition of the natural environment. The assessment covered the production, transport, installation, maintenance, and end-of-life stages. The considerations were conducted using the ReCiPe 2008 methods.

The remaining comparative analyses concerned on onshore and offshore wind farms. Bonou et al. [16] assessed environmental impact of the provision of 1 kWh of wind energy to the power grid. Life cycle assessment analysis of four onshore and offshore power plants was performed using Eco-indicator 99 modelling. Mroziński and Piasecka [17] made comparative analysis of offshore and onshore 2 MW power plant. The research was performed using LCA and Ecoindex-99. Vestas [18] published LCA report of offshore and onshore wind power plants based on the Vestas V90-3.0 MW turbine. This report showed that resource cost of offshore wind turbines is much higher than for onshore power plant. However, electricity gains significantly outweighs the drawbacks. Piasecka et al. [19] performed life cycle analysis of 2-MW offshore and land wind power plants. The study was executed with use of Eco-indicator 99 modelling. Global studies have focused on a comprehensive assessment of the life cycle of large wind turbines [20,21,22] from cradle to grave. SimaPro8 and GaBi software were used for modelling, the results were presented for three areas: human health, quality of ecosystems, and resources use. As a result of these considerations, it was proved that the production phase had the greatest overall impact. Installation, transport, maintenance, and sourcing of raw materials showed a correspondingly lower potential environmental impact.

Among many comprehensive studies of high-power wind turbines, one can find analyses and simulations of the evaluation of selected elements of wind farm construction. Liu et al. [23] draws particular attention to the problem with the development of post-utility first generation of wind turbine blades. In turn, Stavridou et al. [24] made a comparison of wind farm towers composed of: a lattice tower and a tubular one. The result of the study was to demonstrate that the LCA is crucial to assess the actual contribution of these systems of energy production to environmental protection. Therefore, the main objective of the research was the ecological analysis of the life cycle of elements composing an innovative wind power plant.

## 2. Materials and Methods

### 2.1. Object and Plan of Analysis

**Figure 2:**
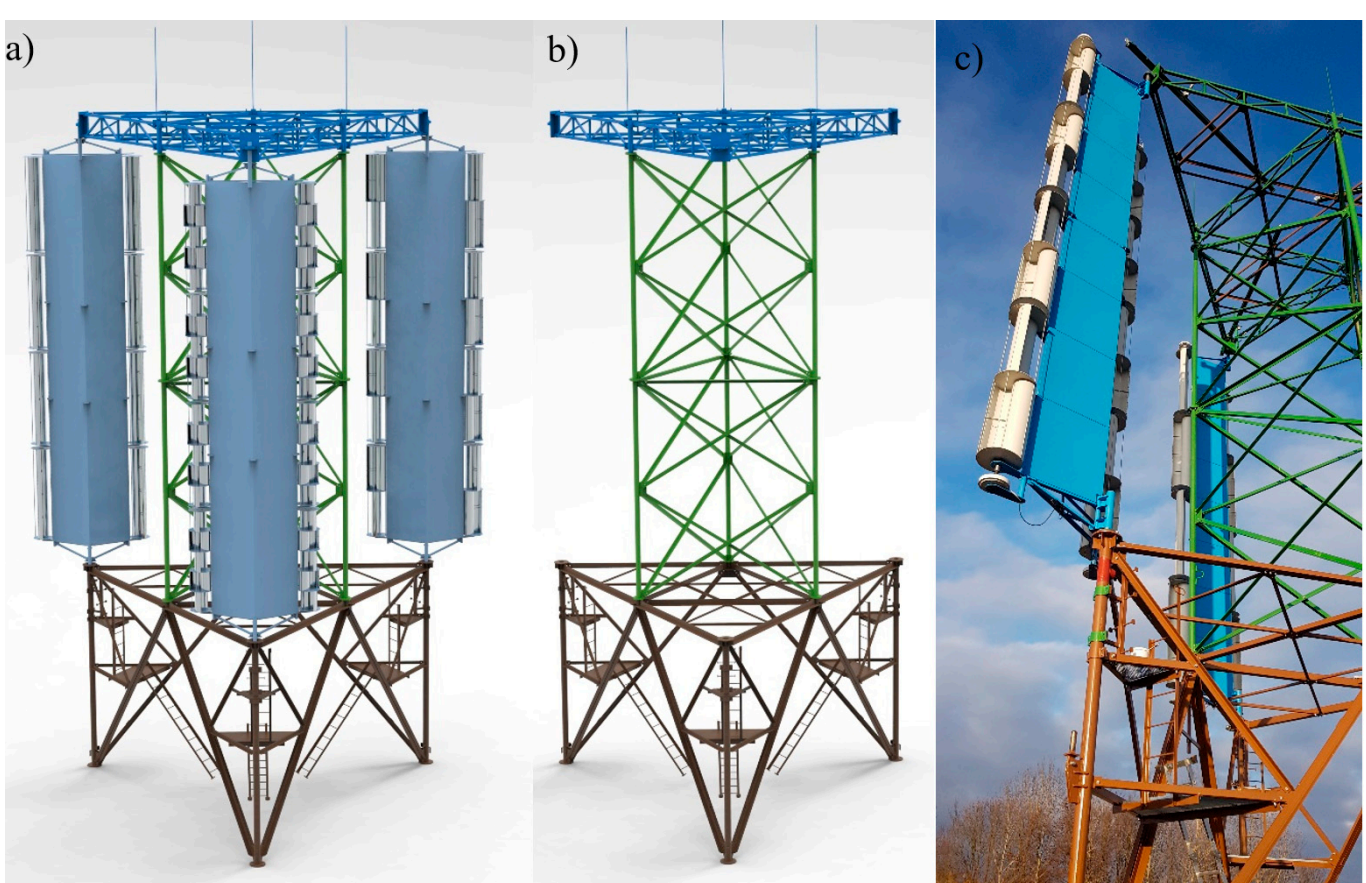
(**a**) wind turbine; (**b**) wind turbine tower; (**c**) wind turbine side view.

The object of analysis was an innovative wind power plant with an output of 15 kW, which is a set of three wind turbines mounted on one support structure (Figure 2). The whole structure consists of elements (tower, turbine structure, rotor, generator, and instrumentation) with a total weight of 11,671.3 kg. The use of an innovative solution of three vertical axis wind turbines on one tower will reduce cost, material consumption and potentially will lower environmental damage.

The LCA—life cycle assessment—was adopted as the main method for assessing potential environmental impacts. According to ISO 14,000 standards, the LCA analysis should include four stages: determination of goal and scope, life cycle inventory (LCI), life cycle impact assessment (LCIA) and interpretation [25,26].

**Figure 3:**
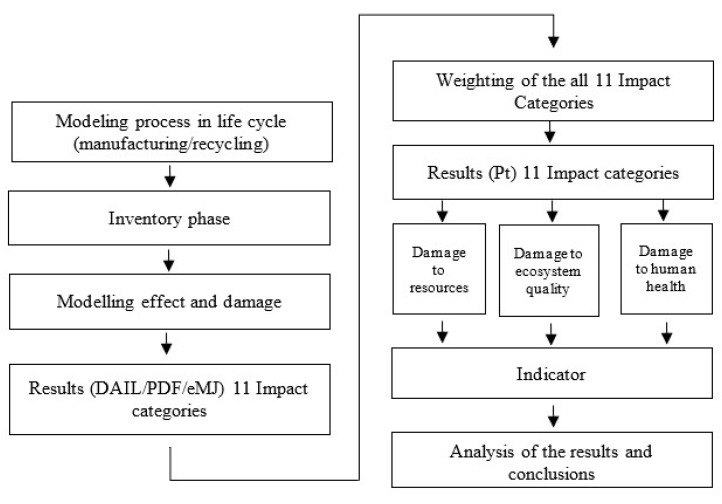
Detailed flow diagram of used methodology.

In accordance with ISO standards, the analysis carried out in this study included four stages. During the first stage, the aim and scope of the analysis were formulated, and their details were approximated in Section 2.2. First step was based on the current state of knowledge and technology analysis. Above mentioned analysis helped to identify the lack of detailed environmental research concerning innovative wind turbines. The basis for formulating the goal and scope was also to collect as much as possible, data of the actual research object of the highest quality. This was possible thanks to the cooperation between universities and the Institute under the POIR.04.01.04-00-0031/18 project. Figure 3 presents order of operations taken during this study. Modeling process, inventory phase, modelling effect and damage and results showed in 11 impact categories were the subject of the first part of the article. Detailed information on the second stage of analyses, i.e., the LCI, is provided in Section 2.3. The third stage consisted of a detailed analysis of the life cycle of an innovative wind power plant. The innovation of the solution occur in modular application of three vertical axis windmills on one tower. All three 5 kW twin-rotor turbines form a 15 kW wind power plant. For the simulation SimaPro 8.4.0 software was used. The basic procedure used for the calculations was the Eco-indicator 99 method. It enabled the assessment of the impact of processes occurring in the life cycle of a wind power plant in three areas of influence: human health, ecosystem quality, and resources. The fourth and, at the same time, last stage (Section 2.5), included interpretation of the obtained results which are presented in Section 3.1, Section 3.2, and Section 4 [27,28,29].

### 2.2. Determination of Goal and Scope

The study examined five elements of wind power plant construction. The research was aimed at a detailed, ecological analysis of the tower, turbine structure, rotors, generators, and instrumentation lifecycle. The research was to describe the existing situation (retrospective LCA). Attempts were also made to model future solutions aimed to develop more environmentally friendly solutions (prospective LCA), which will be presented in future publications. The whole procedure was carried out in accordance with the guidelines presented in the ISO 14,000 standards describing the classic approach to the matter. Analysis was carried out on the life cycle of objects and aspects of their potential negative impact on human and animal health, environmental quality and depletion of natural resources. The life cycles and the scale of the impact of the manufacturing and recycling process were assessed in detail: tower, tower structure, rotors, generators, and instrumentation. The environmental assessment covered eleven impact categories available in the Eco-indicator 99 model: carcinogens, respiratory organics, respiratory inorganics, climate change, radiation, ozone layer, ecotoxicity, acidification/eutrophication, land use, minerals, and fossil fuels. The achieved research results were additionally grouped and compiled in three areas of influence: human health, ecosystem quality and resources [30,31,32,33].

The majority of processes taking place within the life cycles of the examined elements of wind power plant construction take place in Poland. For this reason, the scope of analyses has been referred to the European conditions. The geographical area was considered to be the European territory. The functional unit was defined for one wind power plant with a total capacity of 15 kW. Stages related to storage and transport were excluded from the analysis. The main reason was the difficulty in defining the storage period (it is usually short).

### 2.3. Life Cycle Inventory (LCI)

In order to collect data more simply and precisely, special inventory sheets were prepared. A separate form has been assigned to each individual item. Each sheet included data related to its implementation. The inputs included the main and auxiliary materials. Most of the data were obtained directly from the manufacturer. The study did not use information from the database contained in SimaPro 8.4.0 [27,34,35].

Once the data have been assigned to the relevant construction elements, they have been validated by means of a two-way mass balance. During data completion it was necessary to build their structure systematically. The input and output data streams were balanced in terms of size. This made it possible to group data, which then enabled to obtain a functional unit and reference streams. The input and output stream matrices of all processes are the result of summing data from groups of the same type. The inventory table was obtained by assigning reference streams to them. SimaPro 8.4.0 software requires importing data in the appropriate format to which the inventory table was adjusted. This action allowed to start the third stage of analysis [27,36].

### 2.4. Life Cycle Impact Assessment (LCIA)

Life Cycle Impact Assessment was conducted using SimaPro 8.4.0. (PRé Sustainability, LE Amersfoort, Netherlands) with Ecoinvent 3.4 database. The cut-off level applied to the study was 0.5%. Ecological analysis of the life cycle of elements of wind power plant construction was possible by using the Eco-indicator 99 method. The results of the LCIA stage are presented in Section 3.

The Eco-indicator 99 method belongs to the group of methods modelling environmental impact on the level of the environmental mechanism endpoints. The characterization process is made for eleven impact categories, which fall within three larger groups referred to as areas of influence. The following areas of influence are classified as: human health, ecosystem quality, and resources. The results of the areas of influence indicators are further aggregated into the final Ecolabel through standardization, grouping, and weighting (Table 1) [21,27,37].

Human health is one of the areas of influence in the Eco-indicator 99 method, which consists of six impact categories: carcinogens, respiratory organics, respiratory inorganics, climate change, radiation and ozone layer. By defining an area of impact indicator with endpoints of the environmental mechanism, it is possible to adopt a common unit for all impact categories within the human health framework. Each of them can cause the same type of influence, that is, human and animal health disorders. Murray for the World Bank and WHO developed the DALY scale used at the characterization stage in the Eco-indicator 99 method. This scale is assigned to different diseases. On the scale one end (0 value) is associated with ideal health and the other end is marked 1 and is understood as death [27,38,39].

Within the ecosystem quality there are three impact categories: ecotoxicity, acidification/eutrophication, and land use. The area of impact ecosystem quality is much more diverse and less homogeneous compared to that of human health. The basis of the various processes modelled within its scope is often so different that the common entity is not clearly defined. To solve this problem PAF (potentially affected fraction) unit is converted into PDF (potentially disappeared fraction). In ecosystem monitoring processes, the level of disruption is the most important parameter. In the Eco-indicator 99 method, the disruption rate determines the level of species diversity. Within each impact category, representative species groups are selected. For ecotoxicity (PAF-m2/yr), these were lower terrestrial and aquatic animal species, while the levels of acidification/eutrophication and land use (PDF-m2/yr) were referenced to selected vascular plant species [27,39,40].

Modelling within the third area of influence—resources, consists of a resources, and damage analysis. In Eco-indicator 99 only two impact categories from this area are considered: minerals and fossil fuels. A special damage indicator has been developed, analogous to DALY, PAF, and PDF, which is surplus energy measured in MJ. The extraction of raw materials is permanently leading towards their exhaustion. Depletion of a useful component leads to much more complicated and energy cost extraction methods. It is necessary to predict a decrease in the volume of extracted raw material. Therefore, it is necessary to attempt to model this and include it in the environmental analysis. The model adopted in the research is based on the assumption that a decrease in the quality of a given resource results in a reaction of increased efforts to obtain it from other sources (energy surplus) [21,33,34,35,36,37,38,39,40,41,42]. It is possible to conduct the standardization by referencing the calculated category indicators to the reference information obtained. Standardization is an important tool which is helpful in determining the relative importance of the indicator’s results in relation to a given region (e.g., Europe), a person (e.g., the average inhabitant of Europe), over a given period of time. Weighing procedure is possible thanks to the compilation of LCIA results, which is enabled by prior standardization. On the characterization stage the indicator results are obtained in different units, so it would be difficult to assign specific weighting factors to them and then multiply them. Conversion into a common unit by means of standardization allows for further weighting [32].

In the Eco-indicator 99 method the final step is to obtain the result in the form of Ecolabel [27,43,44]. It is determined by aggregating the previously obtained results, first to three groups and then using them for the final calculation. We meet different types of calculation and analysis variations depending on the purpose and scope of the analysis. Weighting is about determining and assigning the weighting factor (level of importance) to individual impact categories. Weighting factor in the next step is multiplied with standardized indicator results. The weighting process allows us to obtain results in environmental points (Pt). A thousand environmental points are equal to the environmental impact of one European citizen in one year [27].

### 2.5. Interpretation

During the analysis, its completeness was checked with positive results. All key information and necessary data for interpretation were complete. During the analysis, compliance was also checked. The assumptions made, the methods used, the level of analysis, the detail and precision of the data for all the elements of the wind turbine construction under consideration, were in line with the initial aim and scope of the research. The results of the difference analysis for the five construction elements and their detailed interpretation are presented in Section 3 and Section 4 [27,32].

## 3. Results

The results of the life cycle impact assessment (LCIA) are summarized in sections including the Eco-indicator 99.

All results were presented in the unit Pt per 1 p, i.e., the number of environmental points per one piece (11,648.3 kg) of the analyzed wind power plant. One thousand Pt corresponds to the level of potential environmental impact of one European citizen for one year [17,27].

The first step in the area of research using Eco-indicator 99 was a detailed analysis of the eleven impact categories available in this model (carcinogens, respiratory organics, respiratory inorganics, climate change, radiation, ozone layer, ecotoxicity, acidification/eutrophication, land use, minerals, and fossil fuels).

In a second step, the obtained results were grouped into three main areas of influence (human health, ecosystem quality, and resources) and analyzed.

The results were compiled separately for the life cycle and for the recycling processes of the five components of the innovative wind power plant.

### 3.1. Impact Categories

In the early stages of analysis of the impact categories, particular care has been taken to assess which of the eleven categories under consideration may be the source of the greatest number of negative (or positive) environmental impacts over the life cycle of a power plant.

It was noted that the highest level of potential harmful effects on the environment, in the case of all studied objects, is characterized by two impact categories—processes related to fossil fuel extraction (from 2.13 to 125.13 Pt/1 p in the non-recycling method and from −0.45 to 57.74 Pt/1 p in the recycling method) and inorganic compounds causing respiratory diseases (from 5.09 to 166.30 Pt/1 p in the non-recycling method and from 3.23 to 116.91 Pt/1 p in the recycling method). This is due to the high energy needs in the production processes of wind turbines and the directly related, extremely energy-intensive processes of extraction of non-renewable raw materials. During the production process, many inorganic substances are also released into the environment, which are the cause of respiratory diseases, e.g., nitrogen oxides, sulfur dioxide. or particulates (which are a carrier for toxic chemicals, heavy metals, etc.).

On the other hand, two categories with potentially very low environmental impact have also been observed, radiation (0.000 Pt/1 p) and ozone layer (from 0.00 to 0.001 Pt/1 p) (Table 2 and Table 3).

**Figure 4:**
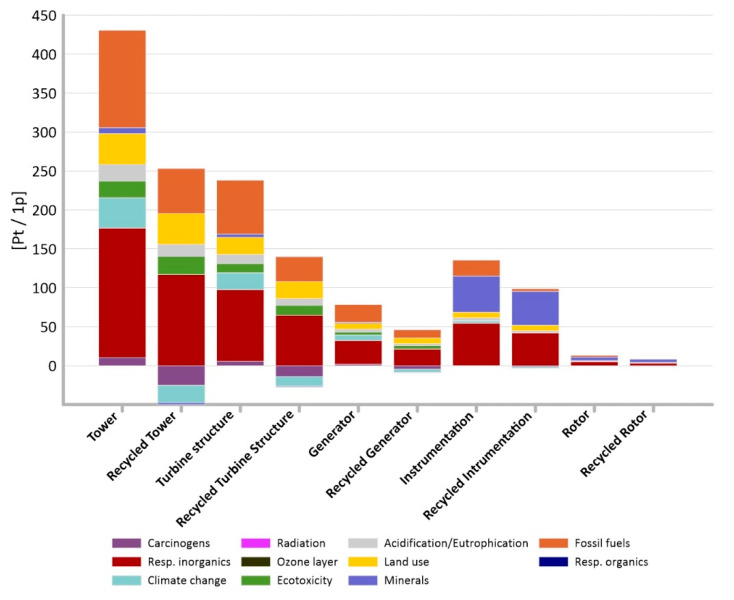
Results of grouping and weighting of individual elements of an innovative power plant for methods with and without recycling.

During the analysis of particular elements of wind turbine construction it was observed that the highest total level of potential negative impact on the environment is characterized by the tower’s lifecycle (total: 430.89 Pt/1 p without recycling method and 203.46 Pt/1 p with recycling) (Figure 4) which represents 57% of the total weight of the structure (Figure 1).

**Figure 5:**
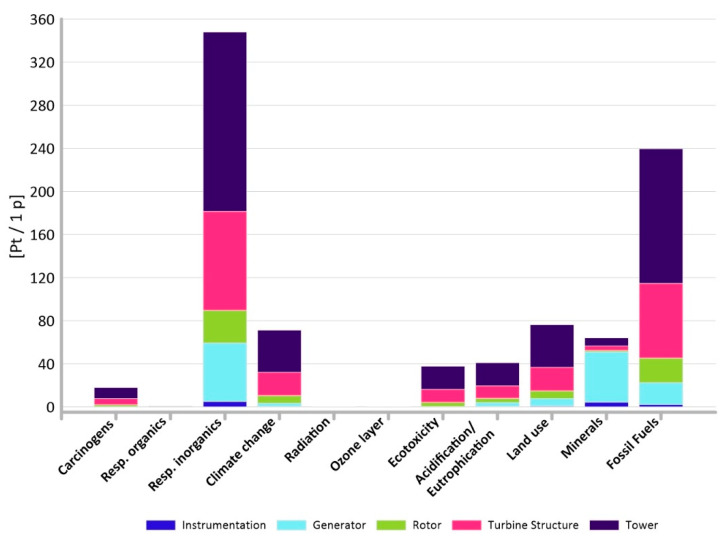
Comparison of results of grouping and weighting of particular impact categories for elements of an innovative power plant—methods without recycling.

**Figure 6:**
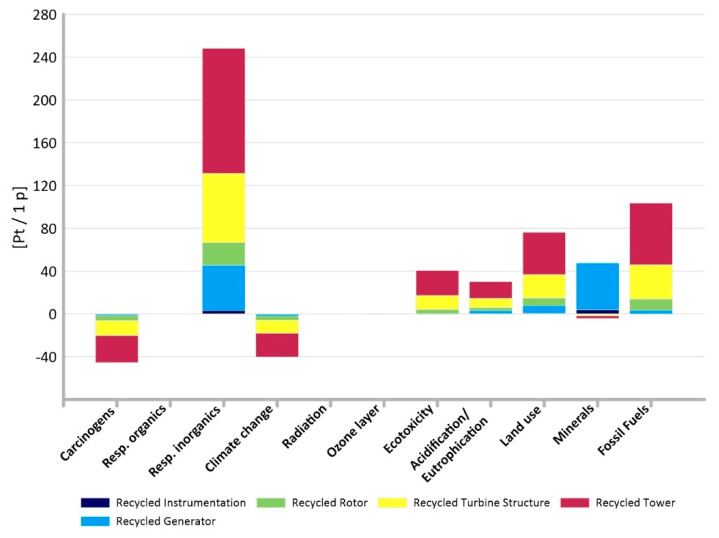
Comparison of results of grouping and weighting of particular impact categories for elements of an innovative power plant—methods with recycling.

It is important not only which elements of the power plant have the greatest impact on the environment, but also which impact categories have a dominant impact on the environment. This is shown in Figure 5 and Figure 6 where respiratory inorganics (total: 347.91 Pt/1 p in the non-recycling method and 248.24 Pt/1 p in the recycling method) and fossil fuels (total: 239.69 Pt/1 p in the non-recycling method and 103.14 Pt/1 p in the recycling method) are noticeably more prominent.

In order to better identify the areas of the life cycle of individual elements of a wind power plant that may have the potentially greatest negative (or positive) impact on the environment, a detailed impact analysis was carried out within the eleven impact categories under discussion, taking into consideration three possible areas of impact on human health, ecosystem quality and resources.

#### 3.1.1. Carcinogens

**Figure 7:**
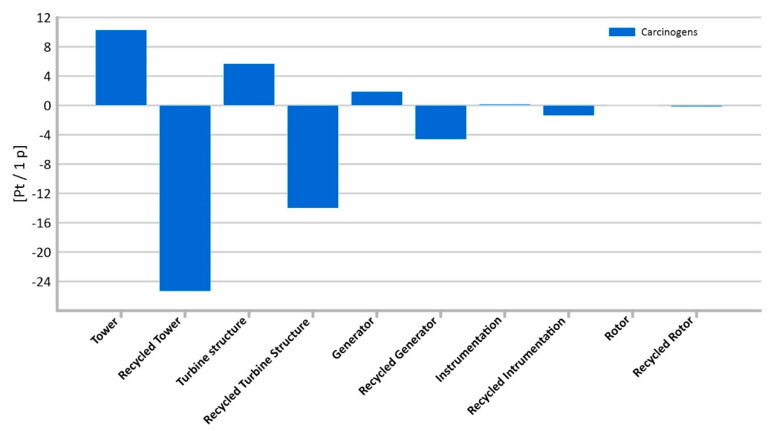
Results of grouping and weighting—carcinogens.

The highest level of potential harmful effects of carcinogenic compounds on human health was recorded for the wind tower lifecycle (total: 10.29 Pt/1 p) (Figure 7). The second largest negative impact of carcinogens compounds on human health was observed for turbine structure (5.69 Pt/1 p). The lowest impact of carcinogens was observed for instrumentation (0.01 Pt/1 p).

The re-use of wind turbine materials can be a potential source of environmental and health benefits. The application of recycling processes to the considered components of a wind power plant may significantly reduce the potential negative impact on the environment in terms of their entire life cycle. The highest level of reduction was recorded for the tower (−25.33 Pt/1 p), turbine structure (−14.01 Pt/1 p), and rotors (−4.62 Pt/1 p).

#### 3.1.2. Respiratory Organics

Among the elements considered during the production of the innovative wind turbine, the highest level of potential emissions of organic compounds causing respiratory diseases is characterized by the tower’s lifecycle (total 0.27 Pt/1 p). It consists mainly of the emissions of some hydrocarbons, related to PVC production processes. Hydrocarbons are the primary component of crude oil. Their emissions to the atmosphere may cause numerous respiratory diseases, including increase of asthma incidence [29,45].

**Figure 8:**
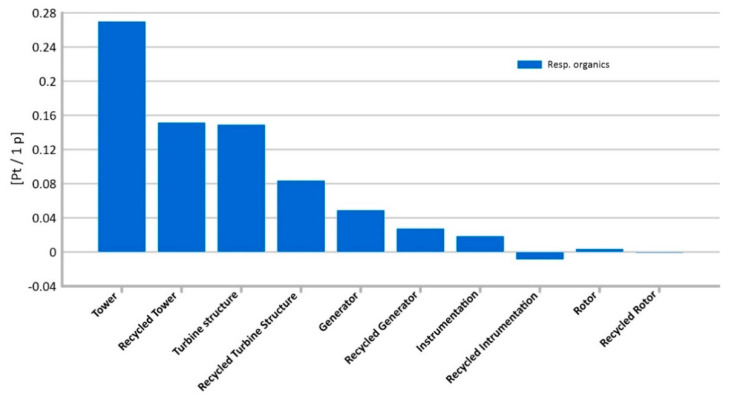
Results of grouping and weighting—respiratory organic.

In comparison with the other analyzed components, the instrumentation and generator life cycles are distinguished by the lowest level of potential dangerous impacts on human health in the impact category under consideration, respectively (0.00 Pt/1 pt and 0.01 Pt/1 p) (Figure 8).

#### 3.1.3. Respiratory Inorganics

**Figure 9:**
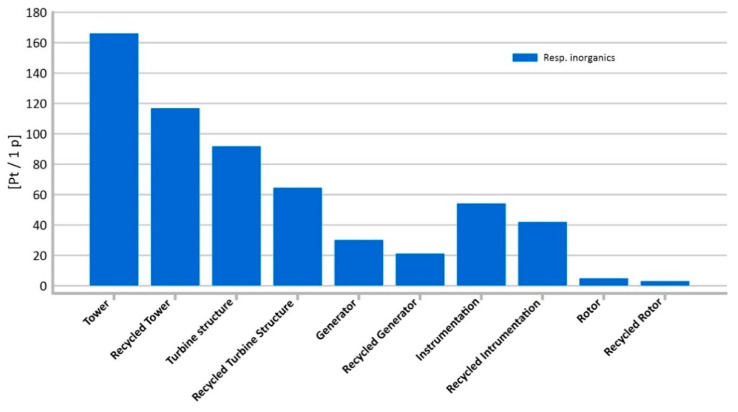
Results of grouping and weighting—respiratory inorganic.

As mentioned before, one of the two impact categories with the highest level of potential harmful effects on the environment is the emission of inorganic compounds causing respiratory diseases. In this area, the most damage to human health can be caused by the tower’s life cycle (total: 166.30 Pt/1 p) (Figure 9). The use of steel in the construction of the tower provides very good endurance properties, but on the other hand, the processes of obtaining the raw material are characterized by a higher level of negative impact on the environment. In the case of this structural element, the use of recycling processes would reduce the negative impacts over the entire life cycle to a value of 116.91 Pt/1 p.

In the category of inorganic compounds causing respiratory diseases, for almost all analyzed types of elements, tower construction, and generators were the second largest contributor to the total amount of potential adverse health effects (from 91.96 Pt/1 p for turbine structure to 54.25 Pt/1 p for generators) (Figure 9).

The application of recycling processes to the analyzed elements of wind power plant construction may significantly reduce the potential negative impact on the environment in the perspective of their entire life cycle, as can be seen in Figure 9.

#### 3.1.4. Climate Change

**Figure 10:**
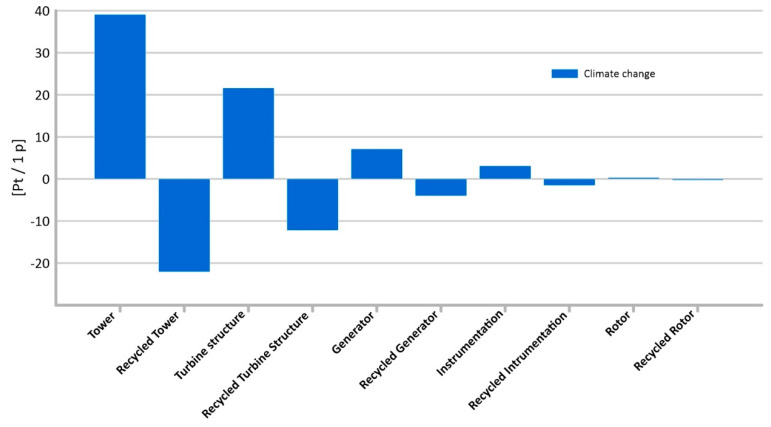
Results of grouping and weighting—climate change.

Nowadays, climate change is one of the key environmental problems worldwide. It is most often considered in the context of global warming caused by greenhouse gas (GHG) emissions. The maximum potential negative impact on the environment of the compounds causing climate change has been recorded within the tower’s lifetime (total: 39.09 Pt/1 p); however, this will change radically when the tower is recycled. The tower’s manufacturing processes require a high energy input, which usually comes from the combustion of conventional fuels. As a result, this not only depletes non-renewable resources, but also reduces the quality of the environment. This, in turn, directly affects many human and animal health problems. The smallest impact on climate change is in the instrumentation life cycle (total: 0.32 Pt/1 p) (Figure 10). The critical compound that shapes the magnitude of the potential negative impact on climate change over the life cycle of all elements considered is carbon dioxide. It plays an important role in the greenhouse effect. Its concentration varies seasonally and with latitude (changes can also be observed locally, especially near the Earth’s surface). The use of recycling as a form of post-consumer management in the manufacture of elements for the construction of a wind power plant reduces the harmful effects of compounds causing climate change, and carbon dioxide in particular, in a life-cycle perspective [29,46]. Recycling allows to reduce the level of potential impact on human health of emissions of climate change compounds for the tower by (61.12 Pt/1 p).

#### 3.1.5. Radiation

Large quantities of materials and energy are used in the production of a wind power plant and the associated fossil resource extraction processes. As already mentioned, energy is most commonly obtained from conventional energy sources, mainly coal. When coal is combusted, dusts and gases are emitted into the atmosphere containing not only harmful substances such as sulfur oxides, nitrogen oxides, mercury vapors, chlorine, fluorine, and heavy metals, but also trace elements which have natural radioactivity. These elements include uranium (U), thorium (Th) and their numerous decomposition products, including radium (Ra) and radon (Rn). Although these elements are chemically less toxic than some other carbon components (such as arsenic, selenium, or mercury), there is a problem, due to the radiation risks [47,48]. The danger of ionizing radiation constitutes a serious risk to human health. In the case of the assessment of an innovative wind power plant, no radioactive emissions were found. The results suggest the existence of potentially negligible doses of ionizing radiation which have not been generated and recorded in the final modelling report. 

#### 3.1.6. Ozone Layer

**Figure 11:**
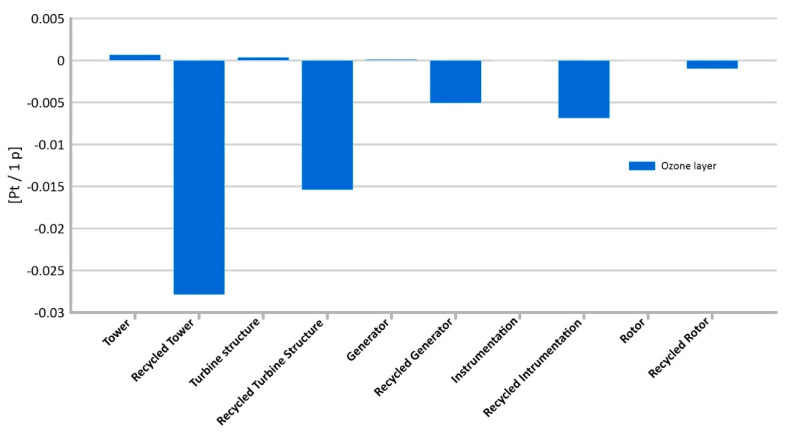
Results of grouping and weighting—ozone layer.

When analyzing the aspects related to the degradation of the environment and, consequently, its impact on human health, the problem of the ozone hole is also important. The stratospheric ozone absorbs part of the ultraviolet radiation reaching the Earth. Certain types of radiation are harmful to living organisms because they can damage cells and the genetic material they contain. For example, cancer can occur in humans and animals. It is therefore appropriate to take measures to prevent the stratospheric ozone (O3) concentration from falling. Comparing the life cycles of the analyzed elements of the wind turbine construction, it can be observed that the tower is characterized by the level of potential emissions of compounds causing the enlargement of the ozone hole at the level of (0.001 Pt/1 p) (Figure 11). For the remaining elements of the wind power plant, no significant impact levels were obtained in this area [49].

On the other hand, the reuse of elements of wind power plant construction may become a potential source of environmental and health benefits. Recycling of all elements of the power plant construction allows to reduce the level of potential impact on human health of ozone layer emissions by 0.03 Pt/1 p for the tower, by 0.01 Pt/1 p for the generator, by 0.01 Pt/1 p for the rotors and turbine structure, and by 0.001 Pt/1 p for the instrumentation.

#### 3.1.7. Ecotoxicity

Among the analyzed elements generated during the production of an innovative wind turbine, the highest level of potential emissions of ecotoxic compounds is characterized by the tower’s lifecycle (total 21.52 Pt/1 p).

**Figure 12:**
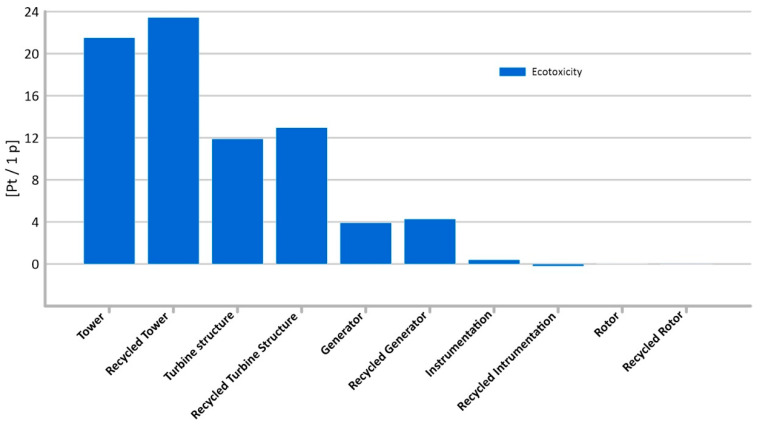
Results of grouping and weighting—ecotoxicity.

Compared to other analyzed elements, the generator and instrumentation life cycle are distinguished by the lowest level of potential hazardous environmental impacts in the category under consideration, not exceeding 1 Pt/1 p (Figure 12).

Recycling is associated with low emissions of ecotoxic compounds (e.g., the ones from chemical reactions resulting from the use of reagents used in such processes).

#### 3.1.8. Acidification/Eutrophication

**Figure 13:**
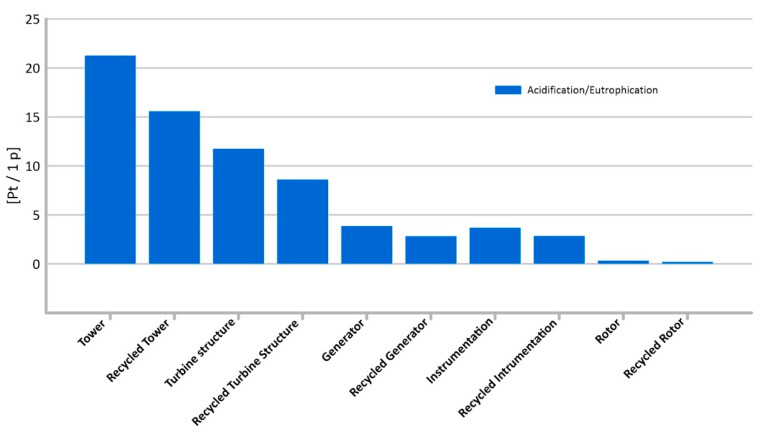
Results of grouping and weighting—Acidification/Eutrophication.

Compounds which have a significant impact on the degradation of the environmental quality may include those which are the cause of acidification or eutrophication. These are present in the life cycle of all elements of wind turbine construction, but the tower (total: 21.28 Pt/1 p) and the turbine structure (15.6 Pt/1 p) may pose a particular threat in this area. In turn, the lowest level of harmful impacts under this impact category was recorded for the instrumentation (0.35 Pt/1 p) (Figure 13).

The use of a recycling process may have a positive impact on the environment, reducing emissions of compounds responsible for acidification or eutrophication by up to (5.68 Pt/1 p) for the tower of a wind power plant.

#### 3.1.9. Land Use

**Figure 14:**
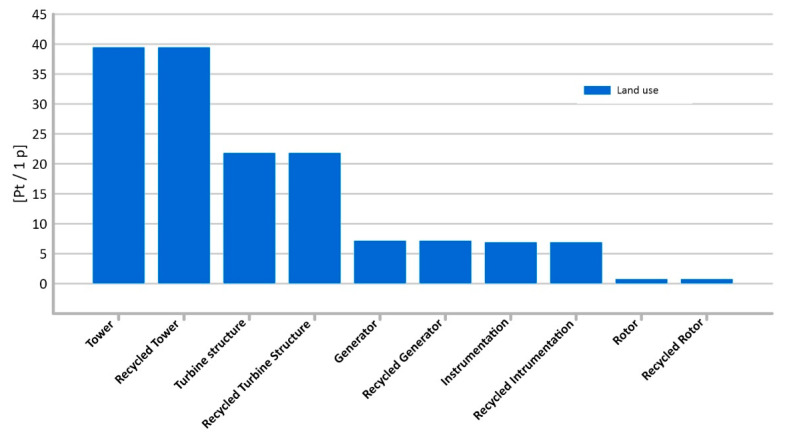
Results of grouping and weighting—Land use.

The different phases of the life cycle of the elements under consideration are inseparable from the need for land use, both in the context of the extraction of fossil fuels and minerals and their subsequent processing. The highest level of potential harmful impact under this impact category is observed for the tower and it has a value of 39.5 Pt/1 p. The lowest potential negative impact in this area is recorded for instrumentation (0.78 Pt/1 p) (Figure 14).

#### 3.1.10. Minerals

**Figure 15:**
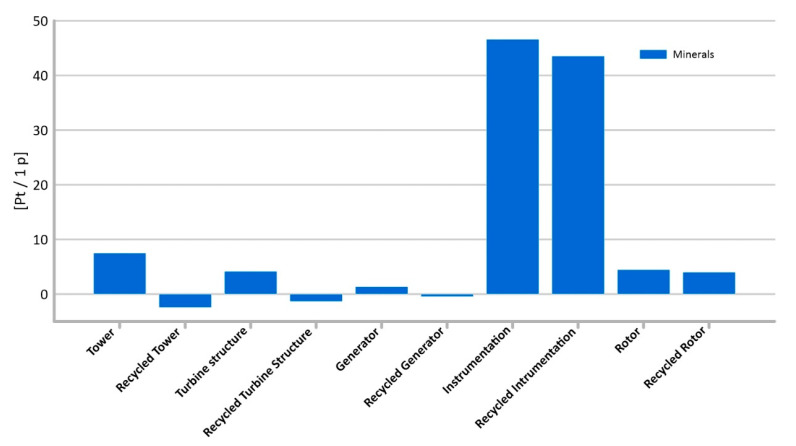
Results of grouping and weighting—minerals.

Every production process, including the building of a wind power plant, is related to the consumption of a certain amount of energy and matter. In order to produce materials and components, it is necessary to extract the relevant raw materials in advance, which also involves a certain amount of energy and auxiliary substances. The highest level of potential harmful impact on the environment of mineral extraction processes has been recorded for the generator’s life cycle (total: 46.6 Pt/1 p), tower (total: 7.5 Pt/1 p). (Figure 15). Therefore, an increase in the impact category is visible in the case of elements made mainly of non-ferrous metals, rare earth alloys, and plastics.

Recycling process of the materials from which the wind power plant is built not only reduces the depletion of minerals, but also significantly reduces environmental degradation. The tower’s recycling processes make it possible to significantly reduce the level of potential harmful effects of the processes involved in obtaining raw materials, in perspective of the entire manufacturing process. The level of reduction of negative impacts varies depending on the type of materials and mass of elements. Recycling may result not only in the complete elimination of harmful effects in the life cycle perspective, but even in an increase in environmental quality.

#### 3.1.11. Fossil Fuels

**Figure 16:**
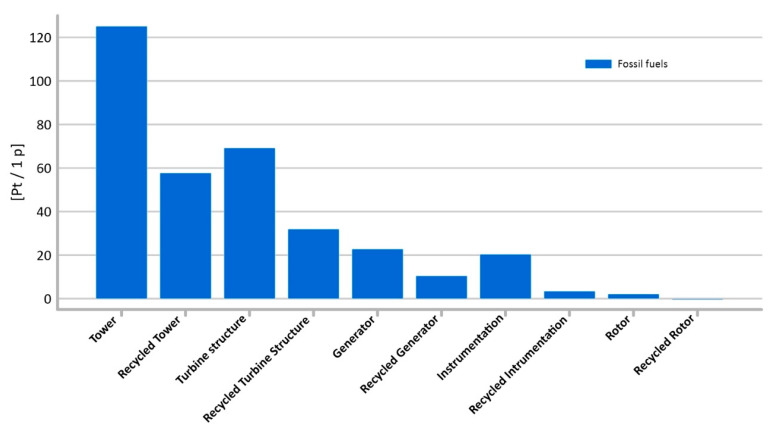
Results of grouping and weighting—fossil fuels.

As has already been mentioned, the main fossil fuels used to produce polymeric materials include crude oil, coal, and natural gas. The processes associated with their extraction result in a significant reduction in environmental quality. The highest level of potential negative impact in this impact category was recorded for the tower’s life cycle (total: 125.13 Pt/1 p), while the lowest—for instrumentation (2.13 Pt/1 p) (Figure 16).

The level of the total harmful impact of the life cycle of the individual elements was influenced primarily by the processes associated with the extraction of crude oil and natural gas. The increasingly rapid consumption of crude oil, natural gas and coal not only leads to the depletion of these non-renewable sources of energy, but their exploitation also brings many problems associated with environmental degradation. The fuel crisis and technological progress have begun an era of oil sands exploitation in the form of opencast mines. The techniques for obtaining these raw materials, due to the properties of the deposit and its location, are more harmful to the environment than traditional extraction of natural gas or liquid oil and lead to even irreversible damage. It is assumed that in an open-pit mine, the extraction of every barrel of oil requires first cutting down the forest, then removing the approximately 2 tons of peat and soil that covers the oil sands, and finally extracting 2 tons of sand itself. Obtaining one barrel of oil from bituminous sand extraction emits approximately three times more CO₂ into the atmosphere than extracting a barrel from traditional deposits in Saudi Arabia. In addition, the water used in the oil extraction process is post-production waste, located near the mine—in the form of a toxic tank. Post-production waste water from opencast oil sands mines contains carcinogenic substances such as cadmium, lead, sulfur, zinc, naphthenic acid, or polycyclic aromatic hydrocarbons, which may cause groundwater pollution [50,51].

Recycling not only reduces the depletion of non-renewable resources, but also significantly reduces degradation of the natural environment. The tower’s recycling processes make it possible to significantly reduce the level of potential harmful effects of processes related to the extraction of fossil fuels, in the perspective of the entire cycle of production of power plant elements (−67,389 Pt/1 p). To a slightly lesser extent, the recycling processes of generator, rotors and turbine structure also make it possible to eliminate potentially negative environmental impacts.

### 3.2. Areas of Influence

Like mentioned earlier, the results of modelling using Eco-Indicator 99 were divided into two groups. The obtained results from eleven impact categories are introduced in Section 3.1, while Section 3.2 presents results which cover three areas of influence (human health, ecosystem quality, resources).

#### 3.2.1. Human Health

In the case of assessed elements, the highest level of potential negative impact on human health is characterized by the tower’s lifecycle (total: 215.95 Pt/1 p) and turbine structure (total: 119.42 Pt/1 p). There is a significantly higher level of harmful effects of the elements, which are mainly made of steel. On the opposite, the instrumentation’s life cycle is characterized by the lowest amount of emissions of substances that can negatively affect human and animal health (5.42 Pt/1 p) (Table 4).

The application of recycling processes to all of the concerned components may result (to a greater or lesser extent) in a reduction of the level of negative impacts on human health in the perspective of their entire production cycle. Thus, recycling processes for human health towers, turbine structure and rotors can reduce negative environmental emissions by around 68%, for instrumentation by around 49%, and for a generator by no more than 32%.

#### 3.2.2. Ecosystem Quality

In addition to substances that affect human health, processes and chemical compounds that cause a reduction in the quality of the environment are also present in the life cycle of elements of wind power plant construction. The negative impact on the environment will, to some extent, also affect human and animal health. Of the assessed elements, the highest level of potential negative impact on the quality of the environment is characterized by the tower’s life cycle (total: 82.3Pt/1 p) (Table 5).

The application of recycling processes to all the components concerned may reduce the level of negative environmental impact and may increase the quality of the environment in the perspective of their entire production cycle. Thus, the processes of instrumentation recycling make it possible to reduce negative environmental emissions by around 19%, in the case of a generator by around 13%, and in the case of other elements of power plant construction by only around 5%.

#### 3.2.3. Resources

Among all of the areas of influence the resources have the largest share in the total impact on the environment within the life cycle of the innovative wind turbine construction elements. Both mineral resources and fossil fuels are the basis of production processes. The highest level of potential negative impact in this area was observed in the tower life cycle (total: 132.64 Pt/1 p) for the turbine structure (total: 75.35 Pt/1 p). The lowest level of negative impact is observed for the instrumentation life cycle (total: 6.59 Pt/1 p) (Table 6).

The implementation of recycling processes for all the elements of the power plant under discussion may result in a reduction of the resources depletion and may increase the quality of the environment in a view of their entire production cycle. Recycling processes are the least likely to reduce negative environmental emissions for the generator by around 30%, for instrumentation by 46%, but by more than 58% for the other elements of the power plant construction.

## 4. Conclusions

Renewable energy sources are considered as the future of energy and hope that they will slow down climate change. Although until now energy has been obtained mainly from fossil fuels, the awareness of public opinion about this method of energy production being a cause of environmental pollution, global average temperatures rise, and depletion of natural resources is increasing [52,53].

The main objective of the study was achieved by conducting an ecological analysis of the life cycle of an innovative wind power plant. The analyses were based on the life cycle assessment (LCA) method and Eco-indicator 99 was used as the calculation procedure. The results were compiled separately for the five construction elements of the wind power plant: generator, instrumentation, rotors, turbine structure and tower.

The highest total level of potential harmful effects on the environment was found in the tower’s manufacturing cycle (Figure 4). Such result confirms conclusions from first part of this study and also corelates with results achieved by Vargas et al. [54] for which nacelle and tower had highest overall negative impact.

The highest potential negative impact on the environment, in the case of all studied objects, was recorded for two impact categories—processes related to extraction of fossil fuels (239.69 Pt/1 p) and inorganic compounds causing respiratory system diseases (347.91 Pt/1 p) (Table 2). Modelling which included recycling achieved values of 103.14 Pt/1 p for extraction of fossil fuels and 248,24 Pt/1 p for inorganic compounds causing respiratory system diseases (Table 3). Such analysis was not possible before grouping and weighting and the influence of inorganic compounds connected with respiratory diseases was hard to identify (Part I). Mroziński and Piasecka [17] in their research of 2 MW wind turbine achieved similar trend. The highest impact was presented by extraction of fossil fuels with value of 58,004 Pt/1 p and inorganic compounds causing respiratory diseases (31, 527 Pt/1 p).

The mining industry and the processing of metals and fossil fuels are the main source of changes in the environment. Especially important are quantities of extracted raw materials and methods used in extraction process. Opencast methods are usually much more destructive than other methods of exploiting deposits. The initial processing of minerals, for which chemical solvents are usually used, also pose a significant threat to human health and environmental quality [51].

In the case of inorganic compounds causing respiratory diseases, the most potential damage to human health in this area may be caused by the tower manufacturing process (Figure 9).

There is an urgent need to change the way in which environmental resources are managed, and experience so far shows that there is great potential for optimizing the use of natural resources. This requires the creation of more sustainable attitudes in the area of production, but also in all economic activities. It is necessary to fundamentally reorganize the entire economy in a way which enable free movement of secondary raw materials between manufacturers and recyclers and other industries [55,56,57,58].

The use of recycling processes can reduce the level of negative impact in the perspective of the entire wind power generation process, by about 53% for the tower, turbine structure and rotors, by about 45% for the instrumentation, and by 29% for the generator.

In obtaining a complete view of the advisability of building a wind turbine, it is reasonable for the environmental losses to be at least compensated by the environmental gains resulting from the construction of such a structure. The first calculable profit is the energy production during the life cycle of the turbine. The second is that a small turbine is an integral part of the advantages of distributed energy, such as the reduction of transfer losses.

The innovative wind turbine was located in a wind favorable area (minimum 1000 kWh/m^2^/year). The active area of the turbines is equal to 75 m^2^ (three turbines of 25 m^2^ each). Assuming total efficiency of converting wind energy into electricity at the level of 20% total production of 375 MWh during its 25 years of life was calculated. The environmental cost in pt/kWh in low voltage installations (<1000 V) for European countries ranges from 10 mPt/kWh in Switzerland to 62 mPt/kWh in Greece. Due to the lack of data for Poland, the calculation example can be taken as for Portugal or Italy, which is 48 mPt/kWh. This allows us to calculate the environmental benefits from the energy produced at a level of 18,000 Pt/1 p. This corresponds to the total environmental cost in the method without recycling (896.47 Pt/1 p) and with recycling (456.03 Pt/1 p). It can certainly be said that this solution is environmentally attractive because the environmental gains are almost forty times higher than the costs. However, it should be noted here that the foundations and transport are not included in the boundary system. The limitation of the boundary system is caused by the need to determine the permanent environmental impact of the power plant. While depending on the location of the turbine, both dimensions, technologies of building the foundation as well as the distance and means of transport are variables.

## Figures and Tables

**Table 1:** Aggregation of data in the Eco-indicator 99 method.

**Impact Category**	**Area of Influence**	**Ecolabel**
Carcinogens	Human health	
Respiratory organics		
Respiratory inorganics		
Climate change		
Radiation		
Ozone layer		
Ecotoxicity	Ecosystem quality	
Acidification/Eutrophication		
Land use		
Minerals	Resources	
Fossil fuels		

**Table 2:** Results of grouping and weighting of environmental impacts during the life cycle of an innovative 15 kW wind turbine -method without recycling (unit: Pt/1 p).

Impact Categories	Tower	Turbine Structure	Rotor	Generator	Instrumentation
Carcinogens	10.29	5.69	1.88	0.17	0.01
Respiratory organics	0.27	0.15	0.05	0.02	0.00
Respiratory inorganics	166.30	91.96	30.30	54.25	5.09
Climate change	39.09	21.62	7.12	3.12	0.32
Radiation	0.00	0.00	0.00	0.00	0.00
Ozone layer	0.00	0.00	0.00	0.00	0.00
Ecotoxicity	21.52	11.90	3.92	0.40	0.03
Acidification/Eutrophication	21.28	11.77	3.88	3.71	0.35
Land use	39.50	21.84	7.20	6.94	0.78
Minerals	7.50	4.15	1.37	46.60	4.46
Fossil fuels	125.13	69.20	22.80	20.42	2.13
Total	430.89	238.28	78.52	135.62	13.17

**Table 3:** Results of grouping and weighting of environmental impacts during the life cycle of an innovative 15 kW wind turbine—method with recycling (unit: Pt/1 p).

Impact Categories	Recycled Tower	Recycled Turbine Structure	Recycled Rotor	Recycled Generator	Recycled Instrumentation
Carcinogens	−25.33	−14.01	−4.62	−1.38	−0.17
Respiratory organics	0.15	0.08	0.03	−0.01	0.00
Respiratory inorganics	116.91	64.65	21.30	42.14	3.23
Climate change	−22.09	−12.22	−4.03	−1.54	−0.30
Radiation	0.00	0.00	0.00	0.00	0.00
Ozone layer	−0.03	−0.02	−0.01	−0.01	0.00
Ecotoxicity	23.44	12.96	4.27	−0.21	−0.06
Acidification/Eutrophication	15.60	8.63	2.84	2.87	0.22
Land use	39.50	21.84	7.20	6.94	0.78
Minerals	−2.44	−1.35	−0.44	43.55	4.01
Fossil fuels	57.74	31.93	10.52	3.40	−0.45
Total	203.46	112.51	37.07	95.74	7.25

**Table 4:** Results of grouping and weighting—human health (unit: Pt/1 p).

Impact Category	Tower	Recycled Tower	Turbine Structure	Recycled Turbine Structure	Rotor	Recycled Rotor	Generator	Recycled Generator	Instrumentation	Recycled Instrumentation
Carcinogens	10.29	−25.33	5.69	−14.01	1.88	−4.62	0.17	−1.38	0.01	−0.17
Respiratory organics	0.27	0.15	0.15	0.08	0.05	0.03	0.02	−0.01	0.00	0.00
Respiratory inorganics	166.30	116.91	91.96	64.65	30.30	21.30	54.25	42.14	5.09	3.23
Climate change	39.09	−22.09	21.62	−12.22	7.12	−4.03	3.12	−1.54	0.32	−0.30
Radiation	0.00	0.00	0.00	0.00	0.00	0.00	0.00	0.00	0.00	0.00
Ozone layer	0.00	−0.03	0.00	−0.02	0.00	−0.01	0.00	−0.01	0.00	0.00
Total	215.95	69.61	119.42	38.48	39.35	12.67	57.56	39.2	5.42	2.76

**Table 5:** Results of grouping and weighting—ecosystem quality (unit: Pt/1 p).

Impact Category	Tower	Recycled Tower	Turbine Structure	Recycled Turbine Structure	Rotor	Recycled Rotor	Generator	Recycled Generator	Instrumentation	Recycled Instrumentation
Ecotoxicity	21.52	23.44	11.90	12.96	3.92	4.27	0.40	−0.21	0.03	−0.06
Acidification/Eutrophication	21.28	15.60	11.77	8.63	3.88	2.84	3.71	2.87	0.35	0.22
Land use	39.50	39.50	21.84	21.84	7.20	7.20	6.94	6.94	0.78	0.78
Total	82.3	78.54	45.51	43.43	15	14.31	11.05	9.6	1.16	0.94

**Table 6:** Results of grouping and weighting—resources (unit: Pt/1 p).

Impact Category	Tower	Recycled Tower	Turbine Structure	Recycled Turbine Structure	Rotor	Recycled Rotor	Generator	Recycled Generator	Instrumentation	Recycled Instrumentation
Minerals	7.50	−2.44	4.15	−1.35	1.37	−0.44	46.60	43.55	4.46	4.01
Fossil fuels	125.13	57.74	69.20	31.93	22.80	10.52	20.42	3.40	2.13	−0.45
Total	132.63	55.3	73.35	30.58	24.17	10.08	67.02	46.95	6.59	3.56

## Data Availability

The data presented in this study are available on request from the corresponding author. The data are not publicly available due to privacy policies.
